# Investigations into Determinants of Blueberry Coating Effectiveness

**DOI:** 10.3390/foods12010174

**Published:** 2022-12-30

**Authors:** David Obenland, Francisco M. A. Leyva-Gutierrez, Tong Wang

**Affiliations:** 1 USDA, Agricultural Research Service, San Joaquin Valley Agricultural Sciences Center, 9611 South Riverbend Avenue, Parlier, CA 93648, USA; 2 Department of Food Science, University of Tennessee, 2510 River Drive, Knoxville, TN 37996, USA

**Keywords:** weight loss, shrivel, decay, waxy bloom, stem end, handling

## Abstract

Coatings have been investigated as a means of slowing weight loss and helping to preserve quality in blueberries but reported results have been inconsistent with the inadequate presentation of the impact of coatings on blueberry appearance. In this study, we compare the ability to limit weight loss, along with the effect on appearance, of several previously studied coatings for blueberries and attempt to identify reasons why coatings have not been more successful in limiting weight loss in blueberries. In a two-year study, coatings were applied either as a spray or a dip, depending on the nature of the coating, and included 1% chitosan (CH) with and without either 1% or 2% oleic acid (OA), 1% Semperfresh (SF), 2% sodium caseinate (SC), and carnauba wax (CAR). None of the coatings reduced weight loss in either year of the study and sometimes enhanced it. CH, CH + OA, CAR, and SF greatly altered the appearance of the berries by removing all or a part of the waxy bloom. SC also did this to some degree but was generally better at maintaining the natural appearance. It was found that coating application did not effectively limit weight loss through either the cuticle or stem end of the blueberries. Loss of the bloom on the blueberry surface, confirmed visually and by scanning electron microscopy, occurred during coating application, but was found to not influence coating effectiveness. Using CH + OA as an example, it was found that increasing the amount of handling during the drying process significantly increased subsequent weight loss relative to blueberries with minimal handling. This indicates that careful handling during the coating process is important for coating success.

## 1. Introduction

Weight loss during storage is a critical factor in determining blueberry quality with 5% to 8% being the level at which blueberries can become unmarketable [1]. This is primarily mediated by a loss of water through the cuticle, although a large proportion also occurs through the stem scar [2]. Cultivar differences exist in the degree of water loss that occurs, and these differences have been found to be linked to both size of the stem scar and cuticle composition [1,3]. Water loss is related linearly to loss in firmness, except when minor water loss occurs where firmness can transiently increase [4,5]. Firmness at harvest and whether the fruit have been damaged, as by the utilization of mechanical harvesting, also impact the degree of softening that may occur in storage. Blueberries that are perceived as being soft when eaten are less preferred by consumers [6], this attribute being of major importance to blueberry quality. In addition, the decline in visual quality in the form of shriveling that accompanies the loss of firmness is considered a major quality defect of blueberries [7]. Minimizing water loss to maintain firmness is, therefore, critical to preserving blueberry quality following harvest.

The cuticular wax coating acts as a critical component to maintain blueberry quality. Chu et al. [8] demonstrated that the removal of the epicuticular wax (bloom) of blueberries exacerbated both weight loss and decay, leading to a decline in sensory and nutritional quality. They further found that fruit so treated had reduced levels of antioxidants and evidence of oxidative damage. While the natural wax layer is thought to be vital to maintaining the quality of blueberries, experimentation has been conducted on the effectiveness of supplementing the natural wax layer after harvest with an additional coating to try to further minimize water loss and slow the rate of senescence. Prior research in blueberries has been most focused on evaluating the performance of polysaccharide-based coatings, such as sodium alginate, chitosan, hydroxypropyl methylcellulose, pectin, or pullulan [9,10,11,12,13]. Caseinates, which are protein-based coatings, have also been studied [9]. Often combination coatings have been utilized to try to enhance coating functionality. Examples of this are high-oleic acid sunflower oil and Aloe vera combined with chitosan to lower water vapor permeability [14,15].

Unfortunately, a great deal of the reported research on blueberries has indicated that the studied coatings have been either inconsistent or ineffective in reducing weight loss for reasons that are unclear. The purpose of this study was to evaluate the performance of a variety of coatings following simulated commercial storage and marketing to obtain a comparison of their effects on blueberry weight loss and quality and, more importantly, to examine potential reasons that may influence their effectiveness. The work also included evaluations on the effect of coating applications on the surface bloom, a characteristic that is indicative of fresh blueberry quality [16] and had received inadequate attention in prior work with blueberry coatings.

## 2. Materials and Methods

### 2.1. Fruit

Varieties Snowchaser and Jewel were picked at commercial maturity from a research field at the USDA San Joaquin Valley Agricultural Sciences Center (USDA-SJVASC) on May 19 and 26, respectively, in season 1 (2020) and season 2 (2021). The field was established in 2016 with 3 m row spacing and 0.9 m between plants in a row. Both irrigation and fertilization were supplied with drip irrigation. Care was taken to pick blueberries that had no visible disorders. Fruit for scanning electron microscopy (SEM) work as well as for the examination of stem end effect were also harvested from the USDA-SJVASC research field. Fruit were obtained in the off-season from a local grocery store to perform a small subsidiary test examining the influence of handling and double coating. Variety could not be obtained in this case.

### 2.2. Coatings

Most coatings were selected due to their prior use in blueberry research. The coatings used were as follows: chitosan (CH, high molecular weight class, ≥75% deacylation, Sigma, St. Louis, MO, USA); Semperfresh (SF, combination of sucrose esters of fatty acids, sodium carboxymethyl cellulose, and mono- and diglycerides of fatty acids, Pace International, Wapato, WA, USA); sodium caseinate (SC, Sigma), and a carnauba wax (CAR) microemulsion (Sta-Fresh 2109, JBT, Riverside, CA, USA). Chitosan was prepared as a 1% (*w/v*) solution in 1% acetic acid using a Tekmar Tissumizer (Cincinnati, OH, USA) to homogenize the mixture, increasing speed as the mixture thickened. When CH was completely incorporated, Tween 20 and glycerol were added to final concentrations of 0.1% (*w/v*) and 50% (*w/w* of chitosan) [9], respectively. In addition, either 1% or 2% of oleic acid (OA) was added in season 1, using high-speed homogenization [17]. SF was made to 1% of the active ingredients (a combination of ingredients as listed above) by dilution in water. A 2% concentration of SC was made in water followed by the addition of glycerol to make a concentration of 50% (*w/w* of SC) and Tween 20 to 0.15% (*w/v*) as reported earlier [9] but substituting calcium caseinate with SC. CAR was used in undiluted form. Controls of water or acetic acid only were included to better estimate true treatment effects. 

### 2.3. Coating Application and Packaging

Coatings were applied either as a dip or, in the case of Semperfresh, both dip and spray at ambient temperature of (23 °C). When applied as a dip the berries were immersed for 15 s, removed from the solution, and then spread out on a screen and allowed to dry under ambient conditions. Care was taken during the coating and drying process to minimize handling and the mechanical impact of the coating process on the fruit. Spraying was accomplished by placing the berries in a single layer on a screen and using a paint sprayer (Wagner, Plymouth, MN, USA) set on a fine spray setting to simulate a potential commercial operation of coating the fruit. Drying in ambient conditions was completed on a screen. In season 1, the fruit were dip-coated a single time while in season 2, the fruit were coated twice to ensure complete coverage and potentially enhance effectiveness. In season 2 the first coating was dried on a screen for 30 min prior to recoating. The effectiveness of recoating and of utilizing additional handling during the coating process was examined in season 1 using CH + OA. In this case, there was a comparison made between minimal handling (normal procedure to apply coating) and one where the fruit were continually turned during drying. After final drying, the fruit from both years were placed into vented plastic clamshells (170 g) and the clamshells placed into boxes capable of holding 12 clamshells. Each clamshell was considered a replication and there were 4 replications per treatment for each storage time.

### 2.4. Storage

In season 1 blueberries were stored at either 3 weeks or 6 weeks at 1 °C and 85% relative humidity in a 7.9 m^2^ storage chamber equipped with a TELSEC 2000 environmental control system (Quest Controls, Palmetto, FL, USA). To better simulate commercial storage and marketing, the storage regimes in season 2 were altered to: (1) 3 weeks at 1 °C, (2) 3 weeks 1 °C + 1 week 10 °C, (3) 3 weeks 1 °C + 1 week 10 °C + 2 d 20 °C. Relative humidity was approximately 95% at 1 °C, 93% at 10 °C and 73% at 20 °C. The same storage chamber was used as in season 1.

### 2.5. Fruit Quality

Percent weight loss was determined by comparing the weights of the individual clamshells at the end of each of the two storage times with the initial values in the experiment. There was no removal of decayed fruit during storage. The appearance of the waxy bloom was rated from 0 to 3, where 0 = perfect, unchanged; 1 = slight loss, acceptable; 2 = moderate loss, not acceptable; 3 = severe loss, not acceptable. Berries that had visible decay, or with softening characteristics of decay, were counted as decays. Those berries that had shriveling that would be objectionable to consumers upon close examination were counted as being shriveled. The shriveling generally occurred at the stem end. Firmness was measured by the use of a FirmTech 2 (BioWorks, Wamego, KS, USA) in season 1 and a FirmTech FT7 (UP Umweltanallytische Produkte GmbH, Ibbenbürn, Germany) in season 2. Both instruments were similar in design and measured the force required in g to cause a 1 mm deflection of the berry. Twenty berries were measured for firmness from each clamshell in both years. After the removal of decayed berries, all the fruit from each clamshell were juiced to measure soluble solids concentration (SSC) and titratable acidity (TA), each clamshell being a replication. The juice was centrifuged at 8100× *g* for 10 min and the resulting clear supernatant was used for the measurements. SSC was determined with a digital refractometer (Atago, Tokyo, Japan) and TA with an automatic titrator (Mettler model T50, Columbus, OH, USA). 

### 2.6. Stem end Contribution to Weight Loss

Eighty berries of similar size and no blemishes were selected from fruit (cv. San Joaquin) that were harvested at the SJVASC when the berries were commercially mature. Twenty berries were used for each of the 4 treatments which were as follows: (1) Untreated; (2) Stem ends covered, no coating; (3) Stem ends not covered, coating; (4) Stem ends covered, coating. Nail polish was used to cover and seal stem ends as in previous work [2] and SC was the coating. Nail polish was applied to the appropriate treatments and allowed to dry, followed by the application of coating if needed. Each berry was individually coated, using forceps to dip the berries into the coating, and carefully placed to dry on Eppendorf tube racks. After drying each berry was individually weighed and reweighed daily for 4 days, after which the fruit were again weighed on day 7. From this data it was possible to estimate changes in weight loss or gain due to SC application from either the cuticle or stem end. 

### 2.7. Scanning Electron Microscopy (SEM)

Coated and uncoated blueberries were carefully packed at the SJVASC in 50-mL Falcon tubes to allow little or no movement of the individual berries, placed into an insulated container with ice packs and sent to the University of Tennessee (Knoxville, TN, USA) by overnight mail. Some of the berries were half-coated to examine the coated/uncoated interface. The surfaces of coated, half-coated, and uncoated fruit were imaged using a Zeiss EVO (Zeiss, Oberkochen, Germany) SEM. Samples were mounted on aluminum holders with double-sided copper tape and analyzed directly (no gold sputter-coating needed) under variable pressure mode (40 Pa) at 200 pA. Images were processed using Microscopy Suite software (version 3.4.3, Gaitan, Pleasanton, CA, USA). All analyses were performed at the University of Tennessee Institute for Advanced Materials and Manufacturing Electron Microscopy facilities (Knoxville, TN, USA). 

### 2.8. Blueberry Bloom Removal Experimentation

Twenty berries, that were free of injury and with substantial bloom, were selected for each of the treatments. Berries with the natural bloom present were used in half of the treatments, while berries for the other half of the treatments had the bloom removed by using Blu Tack adhesive putty (Bostik, Wauwatosa, WI, USA) as previously described [8]. Both sets of berries had the same control and coatings applied: uncoated, CH, SF, SC, and CAR. Coatings were applied by dipping individual berries and then carefully placing them in Eppendorf tube racks to dry. The berries were then transferred to a new rack for storage at 20 °C for 4 days. Weights of the individual berries were taken after the berries had dried and then daily until the end of storage.

### 2.9. Statistical Analysis

Blueberry weight loss and bloom data for seasons 1 and 2 were analyzed by a completely randomized design, using a one-way ANOVA within both variety and storage time using statistical software (SPSS version 24, IBM, Chicago, IL, USA). Similarly, data examining the influence of handling on weight loss were analyzed by ANOVA within storage time and data on the impact of the epicuticular bloom by ANOVA within bloom condition and storage time, both with SPSS. Both of these analyses used a completely randomized design. Mean separations for all analyses in this manuscript were determined using Tukey’s test (*p* ≤ 0.05).

## 3. Results

### 3.1. Effect of Coatings on Weight Loss and Quality

**Figure 1:**
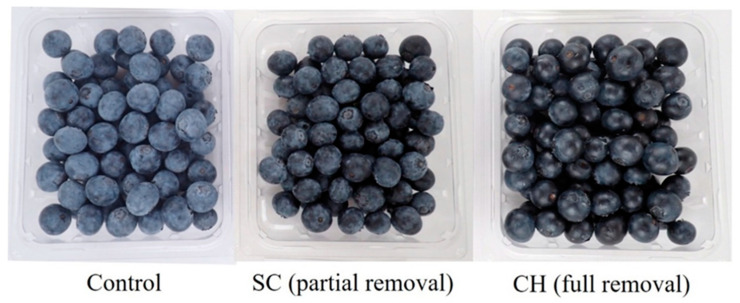
Differential removal of waxy bloom due to coating application. SC being an example of partial and CH of full removal of the waxy bloom.

‘Snowchaser’ and ‘Jewel’ were harvested from the same field in both seasons 1 and 2 and subjected to coating treatments prior to storage. In season 1 the blueberries were stored for either 3 or 6 weeks at 1°C prior to evaluation (Table 1). Presentation of results will focus on coatings and not the associated controls unless the controls indicated that it may have not been just the coating that causes the effect. Weight loss was not different between the untreated control and any coating after both storage times for either variety. For the most part, the waxy bloom was not altered to any sizeable extent by the control treatments throughout the experiments, although ‘Jewel’ after 6 weeks was judged to have enough natural wax removal that marketability might be impacted, indicating that even the control treatments could at times cause substantial damage to the fragile bloom. CH-OA coatings had a strong impact and caused almost complete removal of the bloom, leaving the blueberries to be nearly black in color. Both dip and spray versions of SF caused much less bloom loss and, although the loss was evident, it was in most cases thought not to alter the marketability of the fruit. Two exceptions to this were SF spray following 3 weeks for ‘Snowchaser’ and both dip and spray treatments for ‘Jewel’ after 6 weeks. Examples of the appearance of blueberries with partial or full bloom removal following treatment (applied in season 2) are shown in Figure 1. There was no consistent change due to coating application in decay, shrivel or firmness (Supplementary Table S1).

In season 2, the coating experimentation was altered to include a new storage regime to better approximate handling and marketing. In addition, different coatings were evaluated to find ones that might be more effective than those tested in the prior year. In both varieties, there were no coatings that significantly reduced weight loss below that of the untreated control and the effect of the coatings was often to increase weight loss (Table 2). In contrast to the prior year, there was often seen a negative effect of either the water or acetic acid controls, particularly in ‘Snowchaser’. This may have been due to there being two applications in season 2 rather than one in season 1. As evidenced by the bloom ratings, the waxy bloom was strongly reduced by CH, SF and CAR in both varieties, but in ‘Jewel’ all the coatings negatively altered the bloom to a level that might limit marketability (example photos in Figure 1). The control treatments did not alter the bloom in ‘Snowchaser’ but did occasionally in ‘Jewel’. Fruit coated with CAR often had less decay, and all coatings tended to reduce SSC, but results for shrivel, firmness and TA were inconsistent (Supplementary Table S2). 

### 3.2. Effect of Handling and Additional Coating

An experiment was conducted to examine whether the amount of handling that the blueberries experienced during the coating process or the number of coats applied would alter the coating performance (Table 3). Chitsosan + OA was the coating combination tested. Minimal handling during coating application, which was equivalent to the normal application practice, resulted in a reduction in weight loss from that of the untreated control after 3 weeks but not after 6 weeks. Fruit that received extra handling had increased weight loss above that of the fruit that received minimal handling, although the weight loss was not different than the untreated control for both storage times. The application of an additional coat did not help reduce weight loss.

### 3.3. Influence of Coating on Cuticle and Stem end Weight Loss

**Figure 2:**
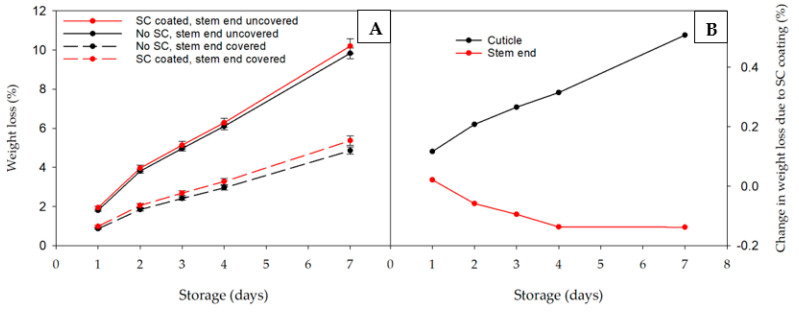
Effect of coating with sodium caseinate (SC) and coverage of the stem end of ‘San Joaquin’ blueberries with nail polish on weight loss during 7 d storage at 20 °C (**A**) and change in weight loss due to SC in either the cuticle or stem end (**B**).

As prior work had demonstrated the importance of the blueberry stem end to weight loss [2], it was of interest to examine the potential effect of a selected coating on this region of the blueberry. SC was chosen as the coating to use based upon its slightly better effectiveness seen in this experiment than the other coatings on preventing weight loss. Blocking the stem end (sealed) of both uncoated and coated fruit dramatically reduced the amount of weight loss by approximately 50%, regardless of the presence of coating (Figure 2A). SC had no effect on limiting weight loss of either sealed or non-sealed fruit. Calculated relative change over time in weight over time due to SC in either the cuticle or the stem end of the fruit is indicated in Figure 2B. Interestingly, SC induced a slight increase in weight loss from the cuticle that linearly increased over time while weight loss from the stem end slightly declined.

### 3.4. SEM imaging of Coated Fruit

**Figure 3:**
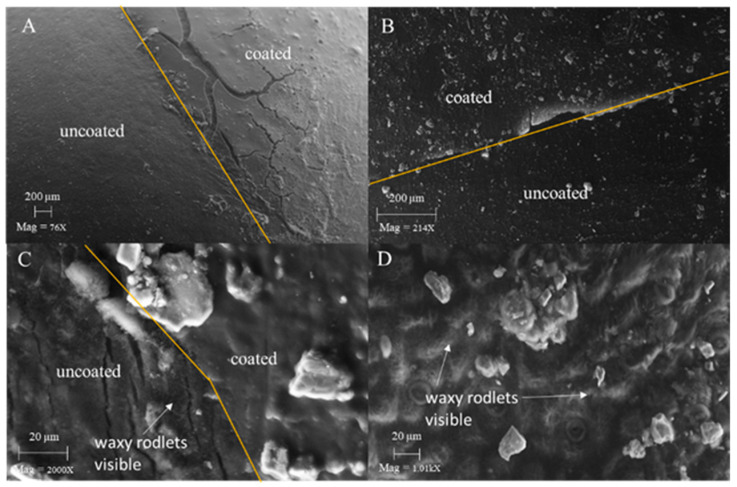
SEM images of blueberries with only one half of the fruit coated (**A**–**C**) or fully coated (**D**) to help better understand coating effects. Carnauba (CAR) coating illustrating the propensity of this coating to crack (**A**). Two magnifications of the chitosan (CH) coating (**B**,**C**) showing its appearance in contrast to the uncoated portion of the fruit. An image of blueberry treated with acetic acid (1%), the control for CH, is shown in a treated area of the berry (**D**).

Coated blueberries were imaged to better understand the poor performance of the coatings in limiting weight loss. SEM images of the various coatings and controls indicated that the coatings were generally continuous and relatively featureless on the fruit surface (images not shown). An exception to this was CAR which tended to crack (Figure 3A). This coating was formulated for citrus fruit and apparently is not appropriate for blueberries even though CAR is an excellent coating in terms of its low water vapor permeability [18]. The representative images in Figure 3B,C were of half-coated fruit to be able to better determine the contrast between coated and uncoated areas. It was hoped that it might be possible to observe additional evidence (beyond darkening of the skin) of the degree that the coatings alter the waxy bloom with the idea that alteration of the bloom may have some role in applied coating performance. In Figure 3C, waxy rodlets that make up the waxy bloom are visible on the uncoated but not the coated side of the fruit. This contrasts with a blueberry dipped in 1% acetic acid, the control for CH, where waxy rodlets are visible throughout (Figure 3D). Since chitosan is transparent then it would have been expected that the wax would have been visible under the coating had the wax been present. 

### 3.5. Blueberry Bloom Removal

Weight loss from blueberries that were coated over the natural bloom or after it had been manually removed was examined to further explore the involvement of loss of the bloom in determining coating effectiveness. Gently wiping the surface of the blueberries with Blu Tack appeared to remove a substantial portion of the bloom as judged by the dark appearance of the berries after wiping. The effectiveness of Blu Tack adhesive for this purpose was previously demonstrated by [8] using SEM imaging. The amount of bloom removed by this process was determined to be an average of 2.9 mg per blueberry as estimated by weight loss. The overall weight loss across treatments was significantly higher (*p* ≤ 0.05) in blueberries with the bloom removed in contrast with those with intact bloom. Except for CAR, which lost significantly less weight relative to the untreated control on days 3 and 4, CH, SF and SC were ineffective in limiting weight loss (Table 4). This was true regardless of whether the bloom had been present prior to coating. 

## 4. Discussion

Experimentation was undertaken in this study to thoroughly characterize the performance of several coatings that had been used in prior research with blueberries and some that had not yet been tried with this commodity. This was used to set the stage for experimentation to better understand why blueberry coatings have often been ineffective in prior research. All the coatings evaluated in this study were ineffective in reducing weight loss, a result that was consistent in both years of the research using two different varieties. In fact, in the second season, weight loss was accelerated by some of the coatings. The only positive results were with CAR when treating individual berries, an impractical procedure commercially. The lack of positive effect in our research on weight loss of chitosan-based coatings agrees with much of the prior research on this coating on blueberries [9,11,14,19,20], although there are some reports that do indicate a benefit [15,21]. The relatively high water vapor permeability of chitosan film [18] likely contributed to the poor or inconsistent results. Although components such as oleic acid are sometimes added to improve water vapor resistance [17], this did not help lower weight loss in the first season of this study where this was tried. SF, a hydrophobic coating in contrast to the hydrophilic CAR, was reported to slow weight loss in blueberries [9], but the results in that study were inconsistent within the duration of the test. In our experiments, SF was not effective in preventing weight loss at any time. Duan et al. [9] also found that calcium caseinate was a poor coating to limit weight loss, the same as our findings with SC. To try to find any coating that would reduce weight loss in blueberries we also tried CAR, a coating with relatively low water vapor permeability [18,22]. This coating also failed, although the fact that the coating was formulated for citrus and not blueberries could have played a part. In addition, unlike in this case, citrus fruit are heat-dried after wax application which may act to soften or partially melt the wax and make its coverage more continuous. Cracking developed during storage may have also limited its effectiveness (Figure 3A). Sodium alginate, pectin, and pullulan, other coatings that have been shown to be of no use in lessening weight loss in blueberry, were not tried [12,13].

It is recognized that coatings have other functions than just lessening weight loss. Since firmness can be reduced by weight loss [5], coatings could potentially make blueberries more firm. Although firmness has been positively impacted in blueberries by coatings in some studies [9,21], often there have been no or inconsistent effects such as those obtained in this study (Supplementary Tables S1 and S2). Our results were not surprising given the poor ability of our coatings to limit weight loss. This was the same situation with shrivel, another quality attribute directly related to weight loss. Decay can cause a severe loss in the marketability of the fruit and, accordingly, this has been an important target of most of the prior studies with blueberry coatings. In the second season, the inhibitory effect of CH on decay was clear in both varieties as has been often noted in various commodities by others [23]. Antioxidant capacity may potentially also be altered by coatings but was not evaluated in this study that focuses on weight loss and external factors due to their greater importance.

The appearance of coated blueberries has not been commented on in almost all prior studies yet is very likely important. Mannozzi et al. [13] mentioned that blueberries treated with sodium alginate or pectin were bluer than the controls. The loss of waxy bloom is responsible for this as was acknowledged in Duan et al. [9]. In this study, we performed visual evaluations for the condition of the bloom to gauge the impact of the different coatings more completely on this quality parameter. Most treatments, including the controls, had some negative impact, although sometimes not to the degree that would likely impact consumer preference. Some coatings, such as CH (with or without OA) and CAR, always were very damaging and caused a complete or near complete removal of the bloom (Table 1 and Table 2; Figure 1). Other coatings, such as SF and SC were more intermediate in effect, causing only a partial bloom removal. A recent study by our laboratory found that partial bloom removal was acceptable to sensory panelists, but full removal was not [24]. This means that a bloom-damaging coating such as CH, despite its benefits in reducing decay, is likely not acceptable for fresh blueberries unless some more gentle means of application can be devised.

After confirmation of the ineffectiveness of the coatings tested in this study, the research moved to experimentation designed to have a better understanding of potential factors for why the coatings were not more efficacious. One of the first areas of the investigation was to examine the impact of handling during the application process as handling was required to both coat and dry the fruit. Using CH + OA as the test coating, it was found that if the amount of handling during the drying process was increased beyond what was normally practiced (minimal handling) the weight loss was significantly greater (Table 3). The results were also similar to CAR (results not shown). This indicates that the methodology of application can influence the success of the coating. The reason for this observation is not known but it is supposed that the coverage of the coating may be compromised by excessive handling. Indeed, SEM observations of blueberries coated with CH, SF, or SC indicated that these coatings formed films with a tendency to flake off (Supplementary Figure S1). Attempts to alter the application method to improve coating performance by spraying instead of dipping were not successful (Table 1). Further experimentation using an air-brush to delicately apply the coating and then drying without handling were also not successful. Surprisingly, it was found that with the CH + OA coating, even the application of an additional coat (using minimal handling during drying) did not lessen weight loss and, in fact, weight loss was even greater than the single coat with minimal handling (Table 3). In one of the few mentions of the mode of coating application for blueberries, Duan et al. [9] noted in their conclusion that dipping may have reduced the effectiveness of their coatings but offered no explanation of why that may have been so. 

The stem end, although small in area relative to the cuticle, was found to be responsible for an average of 40% of the total weight loss in a study that examined a range of highbush blueberry genotypes [2]. To gain insight whether the stem end may be more difficult to coat, and negatively affect coating effectiveness, SC was applied to both blueberries that had the stem end completely blocked using nail polish [2] and control blueberries where the stem end had not been blocked. Eliminating water loss through the stem end eliminated approximately 50% of the total weight loss (Figure 2A), like the findings of Moggia et al. [2]. Same as the research presented earlier in this study, SC did not reduce weight loss in either sets of blueberries. In this case, the handling of the coated blueberries cannot be blamed for the ineffectiveness as each fruit was carefully coated and dried individually. Calculated estimates of the relative effect of SC on both regions indicated the cuticle weight loss was slightly increased over time while that from the stem end does not change much or even slightly declined due to SC application. It appears that, at least with SC, that poor coverage of the stem end was not the reason of the ineffectiveness of the coating in limiting weight loss.

In this study application of coating often led to a visible darkening of the blueberries (Figure 1) due to the loss of epicuticular bloom (Figure 3C). Prior research had shown that removal of the bloom increased weight loss in blueberries [8] and indicated the possibility that damage to the bloom due to coating application may be at least partially responsible for the poor coating performance that we observed. To examine this possibility coatings were applied to natural blueberries (bloom present) and to others that had the bloom artificially removed (Table 4). It should also be noted here that the berries were all individually coated and dried, eliminating the effect of handling. Analysis across treatments indicated that removal of the bloom did result in greater weight loss (*p* ≤ 0.5) as had been previously seen [8], the difference being up to 1.36% by day 3. If damage to the bloom would have played a significant role in the failure of the coatings to prevent weight loss, however, there should have been significant differences between the uncoated and coated blueberries in the group that had the bloom removed as the bloom, in that case, was not a factor. This was not observed, however, indicating that loss of bloom did not influence coating performance.

## 5. Conclusions

Additional confirmation was provided in this study to support previous research indicating the poor effectiveness of coatings to reduce blueberry weight loss. This paper also highlights the fact that coatings alter the appearance by partial or total removal of the waxy bloom. This somewhat neglected aspect needs to be considered in the development and testing of coatings as we have recently shown that consumers prefer blueberries that have at least partial bloom present [24]. Much of this study focused on determining potential reasons why the coatings used in this work performed so poorly to provide means of enhancing the effectiveness of these or other coatings for use for blueberries. Out of all the factors studied only excessive handling during coating was clearly linked to a worsening of coating results. This indicates that careful handling is needed during the coating process, but even with the coating of individual berries where handling was not an issue, we almost never saw that the coatings were effective in reducing weight loss. If coatings are to be used for blueberries it may be that novel formulations, specifically designed for this fruit, are needed for them to be successful. It is also important that consideration be made to ensure that the coatings can be successfully integrated into the commercial packing process that currently exists for blueberries.

## Figures and Tables

**Table 1:** Influence of dip or spray coatings on weight loss and berry bloom rating following 3 weeks or 6 weeks at 1 °C in season 1.

Treatment ^y^	Weight Loss (%)	Bloom (Rating) ^z^	Weight Loss (%)	Bloom (Rating) ^z^
	Loss (%) (Rating ^z^)		Loss (%) (Rating ^z^)	
3 weeks 1 °C	‘Snowchaser’		‘Jewel’	
Untreated	3.47 a	0.0	2.39 a	0.0
Water dip	3.63 a	0.5	3.18 a	1.0
Water spray	3.75 a	0.5	3.23 a	1.0
Acetic acid dip	3.64 a	0.5	3.04 a	1.0
CH + 1% OA dip	3.41 a	3.0	3.26 a	3.0
CH + 2% OA dip	3.52 a	3.0	2.98 a	3.0
SF dip	3.34 a	1.5	3.19 a	1.0
SF spray	3.65 a	2.0	3.40 a	1.0
6 weeks 1 °C				
Untreated	8.74 ab	0.0	6.08 ab	0.0
Water dip	8.43 ab	0.0	3.61 b	2.0
Water spray	7.91 c	0.0	7.96 a	2.0
Acetic acid dip	8.22 ab	0.0	7.32 a	2.0
CH + 1% OA dip	8.63 ab	3.0	6.56 ab	3.0
CH + 2% OA dip	7.70 b	3.0	6.63 ab	3.0
SF dip	7.90 b	1.0	7.49 a	2.0
SF spray	9.60 a	1.5	7.77 a	2.0

^y^ C = chitosan, OA = oleic acid, SF = Semperfresh. ^z^ Waxy bloom coverage: 0 = equivalent to control; 1 = slight loss but acceptable; 2 = moderate loss, not acceptable le; 3 = severe loss, not acceptable. The same letter within the same variety and storage time indicates no significant difference with *p* ≤ 0.05.

**Table 2:** Influence of dip coatings on weight loss and berry bloom rating following simulated storage and marketing in season 2.

Treatment ^y^	Weight Loss (%)	Bloom (Rating) ^z^	Weight Loss (%)	Bloom (Rating) ^z^
	Loss (%) (Rating ^z^)		Loss (%) (Rating ^z^)	
3 weeks 1 °C	‘Snowchaser’		‘Jewel’	
Untreated	2.53 d	1.0	1.83 d	1.0
Water	3.10 ab	1.0	1.98 cd	1.0
Acetic acid	3.18 ab	1.0	2.00 cd	2.0
CH	3.23 ab	3.0	2.50 b	3.0
SF	2.90 bc	2.0	2.10 cd	2.0
SC	2.63 cd	1.0	2.18 bc	2.0
CAR	3.45 a	3.0	2.98 a	3.0
3 weeks 1 °C + 1 week 10 °C				
Untreated	3.35 bc	1.0	2.55 d	1.0
Water	4.05 a	1.0	2.90 cd	1.0
Acetic acid	4.13 a	1.0	2.83 cd	1.0
CH	4.13 a	3.0	3.45 b	3.0
SF	3.88 ab	2.0	2.83 cd	2.0
SC	3.20 c	1.0	3.03 c	2.0
CAR	4.30 a	3.0	4.03 a	3.0
3 weeks 1 °C + 1 week 10 °C + 2 d 20 °C				
Untreated	5.43 c	1.0	3.83 d	1.0
Water	6.78 b	1.0	4.46 bc	1.0
Acetic acid	6.64 b	1.0	4.09 cd	1.0
CH	6.69 b	3.0	4.98 b	3.0
SF	6.56 b	2.0	4.60 bc	2.0
SC	5.71 c	1.0	4.84 b	2.0
CAR	7.69 a	3.0	5.94 a	3.0

^y^ CH = chitosan, SF = Semperfresh, SC = sodium caseinate, CAR = carnauba (undiluted Stafresh 2109). ^z^ Waxy bloom coverage: 0 = equivalent to control; 1 = slight loss but acceptable; 2 = moderate loss, not acceptable; 3 = severe loss, not acceptable. The same letter within the same variety and storage time indicates no significant difference with *p* ≤ 0.05.

**Table 3:** Influence of handling and multiple coats on the effectiveness of a chitosan (CH) + oleic acid (OA) dip on the prevention of weight loss following 3 weeks or 6 weeks at 1 °C using purchased blueberries.

Coating	Weight Loss (%) ^y^	
	3 weeks	6 weeks
Untreated	3.82 ab	7.49 bc
CH + OA minimal handling	2.85 c	6.47 c
CH + OA extra handling ^z^	4.31 b	8.42 ab
CH + OA double coat	4.88 a	9.17 a

^y^ Letters indicate statistical significance (*p* ≤ 0.05) within week. ^z^ Blueberries continually turned while drying.

**Table 4:** Influence of prior epicuticular bloom removal on coating performance during storage at 20 °C.

			Weight Loss (%) ^z^		
Treatment ^x^	Bloom ^y^	Days:	1	2	3
Uncoated	Present		1.44 ab	3.11 a	4.52 a
CH			1.68 a	3.49 a	5.16 a
SC			1.65 a	3.40 a	5.03 a
SF			1.58 ab	3.27 a	4.87 a
CAR			1.39 b	2.90 a	3.82 b
Uncoated	Removed		2.24 ab	4.44 ab	6.48 ab
CH			2.31 a	4.60 a	6.65 a
SC			2.09 ab	4.20 ab	5.84 ab
SF			1.87 b	3.84 b	5.64 b
CAR			2.02 ab	3.87 ab	5.57 b

^x^ CH = chitosan, SF = Semperfresh, SC = sodium caseinate, CAR = carnauba. ^y^ Bloom present or removed by wiping with Blu-Tack prior to coating application. ^z^ Letters indicate statistical significance (*p* ≤ 0.05) within day and bloom removal status.

## Data Availability

The datasets generated in this study are available on request to the corresponding author.

## Supplementary Materials

The following supporting information can be downloaded at: https://www.mdpi.com/article/10.3390/foods12010174/s1, Table S1: Influence of dip or spray coatings on other key quality parameters following 3 weeks or 6 weeks at 1 °C in season 1; Table S2: Influence of dip coatings on other key quality parameters following simulated storage and marketing in season 2.; Figure S1: Example of sodium caseinate flaking visualized by SEM.
