# Supplementary material for: Investigations into Determinants of Blueberry Coating Effectiveness
Source: Foods. 2022 Dec 30;12(1):174. doi: 10.3390/foods12010174 (PMC9818727; doi:10.3390/foods12010174)
Supplement: Supplementary file 1 [file foods-12-00174-s001.zip › foods-2075528-supplementary.pdf]

Supplementary information

Table S1. Influence of dip or spray coatings on other key quality parameters following 3 weeks or 6 weeks at 1°C in season 1.

Treatment[x]	Decay (%)	Shrivel (%)[y]	Firmness (g)[z]
**'Snowchaser'**			
initial	0.0	0.3	214.30
3 weeks 1°C			
Untreated	1.3bc	7.2a	256.17a
Water dip	2.9abc	6.9a	249.23a
Water spray	4.5ab	8.1a	226.45a
Acetic acid dip	0.6c	13.7a	237.44a
CH + 1% OA dip	1.1bc	13.3a	262.68a
CH + 2% OA dip	0.5c	11.0a	257.82a
SF dip	7.1a	6.1a	241.97a
SF spray	1.3c	8.1a	237.15a
6 weeks 1°C			
Untreated	5.6bc	59.0a	197.85a
Water dip	11.2b	30.3cd	182.50a
Water spray	25.3a	26.3d	188.59a
Acetic acid dip	9.6bc	34.0bcd	200.30a
CH + 1% OA dip	1.5c	62.8a	213.96a
CH + 2% OA dip	2.1c	46.4abc	224.36a
SF dip	13.2ab	48.3abc	193.04a
SF spray	10.1bc	51.3ab	196.83a
**'Jewel'**			
Initial	0.0	1.2	223.37
3 weeks 1°C			
Untreated	6.1bc	12.6bcd	224.11a
Water dip	14.2ab	16.8abc	219.62a
Water spray	14.9ab	21.5ab	210.62a
Acetic acid dip	1.7c	26.9a	228.73a
CH + 1% OA dip	12.0ab	23.4ab	247.42a
CH + 2% OA dip	5.8bc	14.2abcd	260.38a
SF dip	13.3ab	6.6cd	237.23a
SF spray	22.9a	4.9d	251.25a
6 weeks 1°C			
Untreated	15.3de	44.6b	211.22a
Water dip	44.9a	9.2c	206.77a
Water spray	36.2ab	11.9c	192.70a
Acetic acid dip	29.3bc	17.8c	198.37a
CH + 1% OA dip	7.2e	71.8a	205.78a

CH + 2% OA dip	6.9e	55.2ab	216.21a
SF dip	18.3cd	58.5ab	207.30a
SF spray	27.1bcd	43.0b	201.60a

[x]CH = chitosan, OA = oleic acid, SF = Semperfresh.

[y]Shrivel: Those blueberries (% relative to total) with moderate or severe shriveling that would be considered not acceptable.

[z]Units of firmness are g/1 mm deflection.

The same letter within the same variety and storage time indicates no significant difference at P=0.05.

Table 2S. Influence of dip coatings on other key quality parameters following simulated storage and marketing in season 2.

Treatment[u]	Decay (%)	Shrivel (%)[v]	Firmness (g)[w]	SSC[x] (%)	TA[y] (%)
**'Snowchaser'**					
Initial	0.00	0.0	158.12	13.0	0.39
3 weeks 1°C					
Untreated	14.78a	2.8b	165.33a	15.1a	0.31d
Water	8.40bc	5.1b	165.02a	14.3ab	0.37bc
Acetic acid	7.03c	6.7b	168.69a	14.4ab	0.34cd
CH	6.63c	6.9b	182.38a	14.0b	0.38bc
SF	13.48ab	5.7b	161.40a	14.7ab	0.41ab
SC	8.08bc	4.6b	170.82a	14.4ab	0.44a
CAR[z]	19.75a	21.2a	176.05a	14.3ab	0.42ab
3 weeks 1°C + 1 week 10°C					
Untreated	24.93b	0.9b	144.59c	15.1a	0.34ab
Water	25.58b	1.4ab	153.79c	14.2ab	0.31ab
Acetic acid	8.00d	3.1ab	160.43c	13.3bc	0.30b
CH	9.60cd	5.8a	185.15ab	13.3bc	0.33ab
SF	17.1bc	0.6b	153.94c	12.7b	0.33ab
SC	14.80bcd	0.5b	163.89bc	13.5bc	0.35a
CAR	41.60a	3.1ab	188.50a	13.5bc	0.35a
3 weeks 1°C + 1 week 10°C + 2 d 20°C					
Untreated	24.50c	2.6a	140.07bc	13.4a	0.32a
Water	34.68b	3.2a	128.09c	13.2a	0.31a
Acetic acid	14.20d	3.6a	123.72c	13.0a	0.29a
CH	13.18d	2.9a	168.00a	13.4a	0.30a
SF	20.75cd	1.6a	128.79c	12.6a	0.30a
SC	16.73cd	1.7a	132.90bc	12.7a	0.34a
CAR	47.50a	2.4a	157.23ab	13.5a	0.31a
**'Jewel'**					
Initial	0.25	1.4	160.71	11.45	0.55
3 weeks 1°C					
Untreated	3.85bcd	4.7bc	121.50b	12.4ab	0.48ab
Water	5.40bc	2.3c	144.80a	11.6cd	0.53a
Acetic acid	0.68d	11.4b	103.98cd	12.6a	0.43bc
CH	8.48b	30.1a	117.74bc	12.5ab	0.37c
SF	2.25cd	7.0b	113.92bcd	11.8bcd	0.52a
SC	23.63a	7.3b	111.18bcd	11.9abc	0.51a
CAR	11.13b	31.2a	103.18d	11.1d	0.52a
3 weeks 1°C + 1 week 10°C					
Untreated	8.30b	5.5bc	125.84bc	12.5b	0.43b
Water	15.45ab	6.0b	136.72ab	11.6c	0.52a

Acetic acid	10.83b	11.2ab	94.37d	13.3a	0.33c
CH	1.03c	16.2a	134.40ab	12.4b	0.42b
SF	12.05b	1.9cd	141.81a	11.9c	0.53a
SC	24.18a	1.5d	120.33c	11.4c	0.54a
CAR	8.33b	6.8b	133.11abc	11.3c	0.55a
3 weeks 1°C + 1 week 10°C + 2 d 20°C					
Untreated	15.28bc	7.5bc	106.62ab	12.4a	0.40b
Water	20.45ab	6.9bc	119.44ab	11.4bc	0.50a
Acetic acid	6.63cd	12.1ab	95.95b	12.7a	0.30c
CH	5.03d	19.5a	108.47ab	12.6a	0.30c
SF	32.20a	5.4bc	138.02a	12.0ab	0.48ab
SC	30.05a	5.6bc	108.49ab	11.2bc	0.48bc
CAR	11.65bcd	4.1c	129.96a	10.9c	0.49ab

[u]CH = chitosan, SF = Semperfresh, SC = sodium caseinate, CAR=carnauba.
[v]Shrivel: Those blueberries with moderate or severe shriveling that would be considered not acceptable.
[w]Units of firmness are g/1 mm deflection.
[x]Soluble solids concentration.
[y]Titratable acidity.
[z]Undiluted JBT Stayfresh 2109.
The same letter within the same variety and storage time indicates no significant difference at P=0.05.

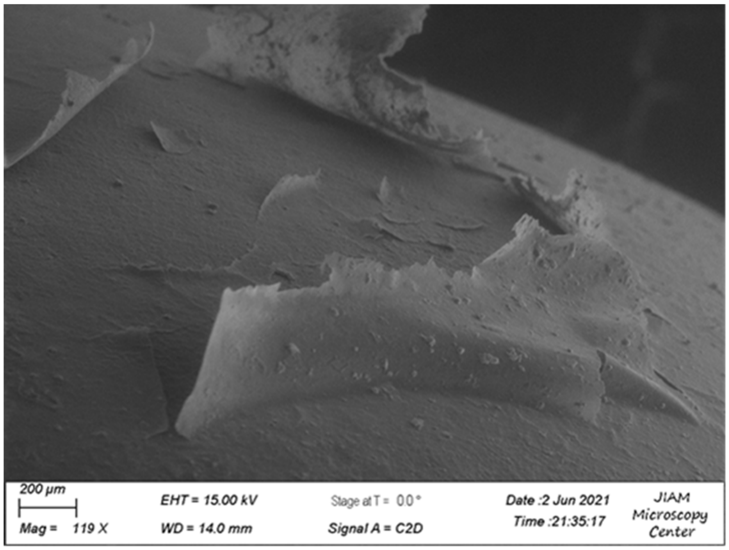

Figure 1S. Example of sodium caseinate flaking visualized by SEM.
